# Behaviour management problems in Finnish children with operated congenital heart disease: a practice-based study

**DOI:** 10.1007/s40368-022-00696-9

**Published:** 2022-03-06

**Authors:** H. Karhumaa, H. Vähänikkilä, M. Blomqvist, T. Pätilä, V. Anttonen

**Affiliations:** 1 Research Unit of Oral Health Sciences, University of Oulu, POB 5281, 90014 Oulu, Finland; 2 Medical Research Centre, Oulu University Hospital and University of Oulu, Oulu, Finland; 3 Infrastructure of Cohort Studies, University of Oulu, Oulu, Finland; 4 Children’s Hospital, Helsinki University Central Hospital, Helsinki, Finland

**Keywords:** Behaviour management problem, BMP, Dental fear, Congenital heart disease, CHD, Child, Caries

## Abstract

**Purpose:**

This retrospective, practice-based study investigates behaviour management problems (BMPs) in dental care among Finnish children with operated congenital heart disease (CHD).

**Methods:**

All the heart-operated children born between the years 1997 and 1999 were identified in the national ProCardio database (*n* = 570). Primary dental care records were requested from this population and were eventually received from 211 patients. Information on gender, diagnosis, number of heart operations and perioperative care were collected from the ProCardio database, and the CHDs were categorised as shunting/stenotic/complex/other defects. Data on BMP/dental fear, oral conscious sedation, dental general anaesthesia (DGA) and past and present caries indices at 6, 12 and 15 years (d/D, dmft/DMFT) were assessed.

**Results:**

Notes on behaviour management problems or dental fear were found in 19% of the study population. BMPs in dental care were more frequent among boys. Children with re-operations, longer post-operative intensive care stay and hospitalisation, and complications had not more BMP than others. Those children diagnosed with syndromes had more BMP often than the rest. Past and present caries experience were significantly associated with BMP, need of oral conscious sedation and DGA. Oral conscious sedation, nitrogen oxide sedation and dental general anaesthesia were used in 17/211, 2/221 and 24/211 CHD patients, respectively.

**Conclusion:**

Dental caries remains a main factor associated with BMP in the CHD population. Need for oral conscious sedation and DGA were rather common. To maintain a good oral health and to avoid development of BMP, CHD children benefit from focus in health promotion and preventive care.

## Introduction

Congenital heart disease (CHD) is the most common congenital defect of a single organ with a prevalence average of 8/1000 live births. Its aetiology remains unclear so far (Reller et al. 2008; Marelli et al. 2014; Moons et al. 2009; Øyen et al. 2009), even if it is associated with certain syndromes, such as Down (trisomy of chromosome 21) (Morales‐Demori et al. 2017). Down and Turner syndromes have a prevalence of CHD up to 50%, being the most common syndromes among CHD children. Down syndrome commonly associates with a shunting defect, most commonly AVSD, VSD or ASD, which cover 80% of the heart diseases among these children (Morales‐Demori et al. 2017; Bergström et al. 2016).

Approximately half of the children with CHD need an operation during the neonatal period or later in childhood. Some children need several re-operations. Not all CHD children need operative care (Verheugt et al. 2017). Those CHD children who are operated, inadvertent recovery or perioperative complications can happen not only after cardiac but also after non-cardiac surgery (Taylor and Habre 2019). Severe, life-threatening diagnosis and heart surgery can be a traumatic experience for the child and the entire family. Post-operative complications often require long hospitalisation. Syndromes are one of the main risk factors for complications (Costello et al. 2018).

Lack of co-operation in dentistry or behaviour management problems (BMPs) is defined as unco-operative and disruptive behaviours, which can delay dental treatment or make treatment impossible (Klingberg et al. 1994). BMP is based on the dentist’s evaluation of the child’s behaviour and includes all levels of difficulties in dental care due to the child’s behaviour. BMP can be, but is not exclusively, a sign of dental fear. In one sample of urban Swedish children, the prevalence of BMP was found to be 10.5%. Among these, 27.3% reported a high level of dental fear, as defined by the dental subscale of the children’s fear survey schedule (Klingberg et al. 1995). On the other hand, 61% of the children with dental fear show BMP (Klingberg et al. 1995). Unlike BMP, dental fear is based on the subjective experience of the patient.

Dental fear associates often with past dental visit experience and painful treatments, but also past hospital visits and admissions have been shown to have an effect (Rantavuori et al. 2004; Suprabha et al. 2011). Childhood traumas, especially emotional ones, associate with dental fear among men, whereas high number of life events seem to correlate with dental fear among females (Hagqvist et al. 2015). Presence of dental fear has a reverse correlation with child’s cognitive capacity and especially with verbal capacity (Blomqvist et al. 2013). Dental fear may lead to avoiding dental care and consequently poor oral health. Poor oral health again promotes fear, which is called Berggren’s vicious circle of fear (Boman et al. 2010). Avoidance of regular dental care and visits only due to pain associate with dental fear (Alshoraim et al. 2018). Importance of good oral health has been recognised; NHS in the UK has recently given guidelines for maintaining good oral health among CHD children, when children with CHD have reported not only to have poorer oral hygiene than other children, and dental fear, but also reduced number of visits to dental care (Hughes et al. 2019). This causes a risk for poor oral health and consequently infective endocarditis. However, literature on BMP affecting oral health among CHD children is scarce.

The aim here was to investigate BMP among Finnish heart-operated children and their need for oral conscious sedation and dental general anaesthesia. Another aim was to study association of BMP and presence of present and past caries experience considering the CHD.

## Material and methods

In Finland, operative care of children with congenital heart disease (CHD) has been centralised in the Helsinki University Hospital. Therefore, study population for the present retrospective study could be collected from the national ProCardio database of Helsinki University Hospital, which includes information of all heart-operated children in Finland. There were 570 Finnish children born between 1997 and 1999, who had had an open-heart surgery at least once in their childhood due to congenital heart disease (CHD) and who had survived up to 18 years of age (2017). The aim was to achieve patient records of these children from primary health care. Dental records were requested from primary health care of the residence municipalities of the participants, who had not moved during their 18 first years of life. History of places of residence among the study population was obtained from the Finnish Population Data Office. Complete dental records were obtained from 211 CHD children, forming the study population. At the time, there were 310 municipalities in Finland and children of the study population lived in 124 of them representing all parts of the country. In this study, the patient records were used as a sole base of information; the participants were not contacted.

Power calculation for sufficient study population was carried out by on-line Sample Size calculator (https://clincalc.com/stats/samplesize.aspx) with 80% power and with alpha-type error at 0.05 and having difference of 15% in incidence of different variables. The sample size of 96 was considered sufficient.

Information of the participants provided by the ProCardio included date of birth, gender (m/f), main diagnosis of the heart disease, presence of syndrome (yes/no), number of heart surgeries, post-operative complications (yes/no), duration of the intensive care stay and hospital stay (days). Complications were classified as follows: tamponade, haemorrhage, neurological problem, infection, permanent need for pacemaker post-operatively and need for re-surgery by other reason than tamponade.

Main diagnoses of CHD were categorised as follows: (1) shunting disease [ventricular septal defect (VSD), atrium septal defect (ASD), atrium ventricular septal defect (AVSD), patent ductus arteriosus (PDA)]; (2) stenotic disease: aorta stenosis (AS), coarctation of aorta (CoA) and pulmonary stenosis (PS); and (3) complex disease: univentricular heart syndrome (UVH), tetralogy of Fallot (TOF) and transposition of great arteries (TGA). These defects comprised 90% of the study population. The fourth category (other defects) comprised those with rare multiform congenital heart diseases.

From patients records of primary health care in the municipalities, data on BMP were compiled based on notes in the dental records concerning dental fear by dental professionals. BMP was considered to exist if comments on BMP or dental fear were observed in the records on two or more visits between the ages of 6 and 18 years expressing disruptive behaviour that delayed treatment or rendered treatment impossible. Here, only term BMP was used. Additionally, use of oral conscious (yes/no) and nitrous oxide sedation (yes/no) in dental care as well as dental general anaesthesia (DGA) (yes/no) were registered if they were mentioned at least once in patient records. Dental caries prevalence was registered as dt/DT and dmft/DMFT values from the point of time when the participants were in the first, fifth and eighth grades (6, 12 and 15 years, respectively), when dental examination is compulsory for everyone in Finland. The number of examinations by dentists was recorded as well (*n*).

### Statistics

The study population was described as frequencies and proportions. For contingency analysis Chi-square test was used. Kruskal–Wallis and ANOVA tests were used to compare the means of caries indices and presence of BMP, DGA or heart surgery-related post-operative complications. Differences between the groups were considered statistically significant with *p* values < 0.05. All analyses were conducted using SPSS version 24.0 (Chicago, Illinois, USA).

### Ethics

The Finnish ProCardio database comprises the data of all heart-operated children in Finland, including date of birth, gender, diagnosis, date of heart surgery and post-operative conditions. Survival data is updated regularly. Parental permission has been achieved to use the patient data in scientific research. The Helsinki University Hospital District has given a favourable ethical opinion on the study (165/13/03/03/2012). The permission for this registry study was received from the Northern Ostrobothnia Hospital District (248/2017). Some municipalities required a separate study permission from them.

We collected data of all heart-operated CHD children in Finland, who were born between 1997 and 1999, and were operated due to CHD at least once during their childhood. Although operated in a single centre, the children were living in different parts of the country. Residential history of the study population was obtained from the national Population Register Center; with that information, patient records were collected from children’s primary health care centres. The permission of the register keeper (in this case, municipalities) allowed us to collect register information (diagnosis, indices). All analyses were carried out anonymised.

## Results

In the study population (*n* = 211) more than half were boys, 13.7% had a syndrome. Among those with a syndrome, significantly bigger proportion (72.4%, *p* = 0.03) were boys (Table 1). Mean number of heart operations was 1.3 (min 1, max 9, SD 0.9). Number of post-operative hospitalisation days varied from 2 to 101, and mean was 16.4 (SD 16.5). Mean number of days in post-operative intensive care was 4.2 (SD 6.7, min 1, max 71).

**Table 1:** Distribution of participants according to CHD, gender as well as prevalence of BMP and use of oral conscious sedation, nitrogen oxide sedation and dental general anaesthesia

CHD type	Gender	*n* (%)	Syndrome *n* (%)	BMP *n* (%)*	Oral conscious sedation *n* (%)*	Nitrogen oxide sedation *n* (%)*	Dental general anaesthesia *n* (%)*
Shunting defects	Girls	48 (50.5)	8 (16.7)	9 (18.8)	4 (8.3)	0 (0.0)	6 (12.5)
	Boys	64 (53.8)	21 (32.8)	17 (26.6)	9 (14.0)	2 (3.1)	10 (15.6)
Stenotic defects	Girls	9 (9.5)	0 (0.0)	1 (11.1)	0 (0.0)	0 (0.0)	0 (0.0)
	Boys	22 (18.5)	0 (0.0)	2 (9.1)	1 (4.5)	0 (0.0)	0 (0.0)
Complex defects	Girls	24 (25.3)	3 (12.5)	5 (20.8)	1 (4.2)	0 (0.0)	3 (12.5)
	Boys	23 (19.3)	5 (21.7)	5 (21.7)	2 (8.7)	0 (0.0)	4 (17.4)
Other	Girls	10 (10.5)	1 (10.0)	0 (0.0)	0 (0.0)	0 (0.0)	0 (0.0)
	Boys	11 (9.2)	0 (0.0)	1 (9.1)	0 (0.0)	0 (0.0)	1 (9.1)
Total	Girls	91(43.1)	12 (13.2)	15 (16.5)	5 (5.3)	0 (0.0)	9 (9.5)
	Boys	120 (56.9)	26 (21.7)	25 (20.8)	12 (10.1)	2(2.5)	15 (2.6)
*p*							
Total		0.273	0.003	0.113	0.174	–	0.392
Girls			0.578	0.445	0.587		0.342
Boys			0.003	0.257	0.367		0.363

*Difference between the genders and CHD types was not statistically significant

Notes on BMP or dental fear in the patient records on at least two occasions between 6 and 18 years of age were found in 19.0% of the CHD children, somewhat more frequently among boys than girls. BMP was significantly associated with the presence of a syndrome; in 46.2% of the syndromic boys and 33.3% of the girls. BMP was registered fairly frequently among children with shunting and complex heart defects (Table 1).

The number of surgeries or hospitalisation or intensive care days was not associated with BMP. One-fourth (25.7%) had a post-operative complication at least once, 60% of whom were boys. No distinct association was discovered between post-operative complications and prevalence of BMP (Table 2).

**Table 2:** Association of BMP and stay in hospital in days after heart surgery and proportion of those with complications during heart surgery

BMP	Mean number of heart surgery (SD)	*p*	Mean hospitalisation days due to one heart surgery (SD) (*n* = 180)	*p*	Mean number of days in intensive care unit after one heart surgery (SD) (*n* = 160)	*p*	Complication during heart surgery (%) (*n* = 55)	*p*
Yes	1.3 (0.7)	0.478	13.3 (12.5)	0.558	3.8 (3.6)	0.690	30.0	0.490
No	1.4 (0.9)		11.9 (11.5)		4.3 (7.2)		24.7	
Total	1.3 (0.9)		12.2 (11.6)		4.2 (6.7)		25.7	

BMP was significantly associated with the present and past caries experience at 6, 12 and 15 years. Children with BMP had significantly more frequently oral examinations by a dentist (mean 9.5 [SD 3.7]) than those without (mean 7.6 [SD 3.7]) (Table 3).

**Table 3:** Association between caries incidence and prevalence and BMP, post-operative complications during heart surgery and DGA at least once in childhood

	dt/dmft 1st grade (SD)	*p*	dt/dmft 5th grade (SD)	*p*	DT/DMFT 6y (SD)	*p*	DT/DMFT 12y (SD)	*p*	DT/DMFT 15y (SD)	*p*
BMP							1			
Yes	1.3 (2.3)/3.9 (4.8)	0.020*/ < 0.0001*	0.2 (0.9)/4.5 (5.3)	0.984/ < 0.0001*	0.4 (1.0)/0.6 (1.3)	0.033*/0.012*	1.5 (1.0)/3 (1.9)	0.038*/0.032*	1.2 (2.1)/3.2 (3.5)	0.014*/0.001*
No	0.6 (1.3)/4 (2.3)		0.2 (0.7)/2.1 (2.8)		0.1 (0.5)/0.2 (0.6)		0.3 (0.6)/0.8 (1.3)		1.6 (1.3)/5 (2.4)	
DGA							2			
Yes	1.9 (2.7)/5.2 (5.6)	< 0.0001*/ < 0.0001*	0.1 (0.5)/6.1 (6.0)	0.622/ < 0.0001*	0.4 (1.6)/0.8 (1.6)	0.050/0.002*	1.5 (0.7)/4 (1.9)	0.251/0.076	0.9 (2.2)/3.4 (3.6)	0.604/0.005*
No	0.6 (1.3)/5 (2.4)		0.2 (0.7)/2.1 (2.8)		0.1 (0.5)/0.2 (0.6)		0.3 (0.7)/0.8 (1.4)		1.7 (1.3)/7 (2.6)	
Complication during heart surgery										
Yes	0.6 (1.5)/2.2 (4.0)	0.523/0.507	0.3 1.0)/2.7 (4.1)	0.107/0.757	0.2 (0.7)/0.2 (0.7)	0.865/0.763	0.4 (0.8)/0.9 (1.4)	0.191/0.899	0.9 (1.8)/2.0 (2.7)	0.362/0.662
No	1.8 (1.6)/8 (2.8)		0.2 (0.5)/2.5 (3.3)		0.2 (0.6)/0.3 (0.8)		0.3 (0.6)/0.9 (1.5)		1.7 (1.2)/8 (2.8)	
Total	1.8 (1.6)/9 (3.2)		0.2 (0.7)/2.6 (3.6)		0.2 (0.6)/0.3 (0.8)		0.3 (0.7)/0.9 (1.5)		1.7 (1.4)/9 (2.8)	

*Statistical significance

Only 8.1% among the participants had received oral conscious sedation at least during one dental visit, most of whom were boys (70.6%). Nitrous oxide sedation was seldom used (1.0%), whereas DGA was used (11.4%) more frequently specifically among those with shunting or complex CHD (Table 1). dt/DT was significantly associated with DGA at 6 years, whereas dmft/DMFT was statistically significantly associated with DGA in all age groups except at 12 years (Table 3).

## Discussion

This retrospective practice-based work adds to limited literature on BMP among operated children with congenital heart disease (CHD). Dental anxiety and BMP can hinder a child getting dental care, which is essential for good oral health and their systemic well-being. BMP was as common among CHD children as previously reported with children having any chronic disease (Rajavaara et al. 2018) or disabilities (Kankaala et al. 2019). BMP was here more frequently registered for boys than for girls and was associated with shunting defects and the presence of a genetic syndrome. Interestingly, no association between BMP and the number of heart operations, intensive care stay or hospital stay was found. However, present and past caries experience associated with BMP.

Syndromes are common among children with CHD, which was true here as well, when 14% of the study population had some genetic syndrome. The most common syndrome among these CHD children was Down syndrome, but also Turner’s or Catch22-syndromes were present in the study population. Children with Down syndrome are the largest group of pediatric patients with dental special needs (Wang et al. 2012). Down syndrome is also associated with gender being more prevalent among boys. Genetic syndromes are often associated with cognitive development delays, i.e. children with Down syndrome have prominent delays in nonverbal and verbal communication and short-term memory (Chapman and Hesketh 2000). Presence of dental fear has a reverse correlation with child’s cognitive capacity and especially with verbal capacity (Blomqvist et al. 2013). Over presentation of boys among those with BMP in this study population may at least partly be explained by a large proportion of children with syndromes. Unfortunately, children with any cognitive problems and special needs often are at risk for having increased dental treatment need. This may be serious for children with CHD.

It has been reported earlier that frequent visits to the medical doctor and hospitalisation periods may influence child’s behaviour in the dental office and cause him to be reluctant and suspicious to potentially painful dental procedures (Suprabha et al. 2011). However, in our population, the frequency of BMP was not increased in patients with more re-operations or with those who needed a longer post-operative recovery in the intensive care or in the hospital. Yet, earlier experiences in medical care must be assessed by the dental professionals in the treatment planning. Collaboration between the dental and medical team would be beneficial for the child as well as collaboration between primary and specialised dental care (Hughes et al. 2019).

Dental caries was associated with BMP here. Maintaining good oral health and avoiding risk of infections specifically infectious endocarditis due to oral conditions, most often dental caries, when children are concerned, are of utmost importance for children with CHD. These children should be considered of being at increased risk of dental caries and consequently they should be monitored regularly. In our study cohort, the fearful children had more appointments for examinations than the rest, which shows that dental professionals at least aim to emphasise importance of dental visits of CHD children with behaviour management problems. Individual caries controlling plans with preventive means should indeed be implemented (ICCMS 2014, https://www.iccms-web.com/content/iccms-usage/clinical-practice).

More than one in ten of CHD children in the study group had a dental general anaesthesia (DGA) at least once during their childhood years. Children with any chronic disease seem to be referred more easily to DGA compared to healthy ones (Rajavaara et al. 2017). DGA is often necessary, but the primary goal should be to treat children conventionally. It has been shown that after dental general anaesthesia, the problems during dental visits in the general practice remain. Therefore, dentists in general practice, especially those treating children, should have basic skills of dealing with BMP (Kankaala et al. 2019).

Several reasons can lead CHD parents to feed their children more frequently any desired foods, often rich in fermentable carbohydrates. Even short exposures to sucrose may cause increased risk for dental caries if the oral condition favours dental caries (Anttonen et al. 2012). This may explain caries risk among the CHD children here, and may have been a reason for BMP. To the benefit of their child, parents should be advised of the causative role of sucrose in the development of caries and emphasise the possible serious consequences of caries (Paes et al. 2006). Parents should also be given means to support their child’s oral health in home care. This would help in preventing dental fear as well.

Even, if use of dental records can be questioned for scientific works, a practice-based study like this one can be considered as valid as a clinical study in assessing the prevalence of caries and the number of dental visits (Hausen et al. 2001). Using register data can be cheaper and faster than collecting data through clinical trials (Pavlovic et al. 2009). Dentists in Finnish primary health care are paid according to the procedures, so it is unlikely they would miss registering them. In recent years also the importance of ICD-10 diagnoses codes has been emphasised among dentists. BMP noted in patient files as dental fear can be considered quite reliable, too, but to be certain, we included here only those cases with dental fear registered at least twice in the patient records. At present, a Finnish dentist is allowed to diagnose a patient with a dental fear diagnosis (ICD10 F40.2).

The register keeper or generally the chief dentist/medical officer in the municipality can give permission to use patient records, which made the process fairly simple at the time of data collection in Finland. Achieving patient files was, however, quite laborious, and not all municipalities responded.

The concentration of operative CHD care in Helsinki University Hospital was an advantage. Thus, our study population represents the whole country. Another strength is that practically all children in Finland receive dental care in primary health care units, despite their social status, and therefore, their patient files were available. As for limitations, the strict rule of BMP with at least two notices of dental fear might underestimate the real number of children with BMP. In future, studies are needed on this topic and also by using forms investigating dental fear, i.e. FIS, PCPD, which have been validated in Finnish (Keränen et al. 2021). Finally, not all municipalities responded to our data requests. Use of dental fear scales (MDAS, DVAS) would have given more information on dental fear as well as oral health-related quality of life (for instance, OHIP-14) (Rantavuori et al. 2004; Humphris et al. 1995, 2000; Luyk et al. 1988), which can be considered shortcomings. Use of dental fear scales, is most beneficial in planning and monitoring the outcome of treatment of fearful dental patients. These facts should be kept on mind when designing new research on this topic.

Oral health and avoiding serious consequences of oral infections are essential for general health of children with CHD. These children need special attention in many respects. Dental professionals should be trained and motivated for using cognitive behaviour treatment (CBT)/BT in their everyday practice, specifically for these children. Treatments due to heart condition seem not to be associated with BMP, but instead dental caries remains the main factor associated both with BMP and DGA among children with CHD needing heart surgery. Oral health should be kept in mind while planning medical care and impact of medical history should be taken into consideration in dental care. Collaboration and information between dental and medical officers would be beneficial for the child.

## Conclusion

Considering the limitations of the present study the following conclusions can be made:

- BMP was registered in almost every fifth child with operated congenital heart disease (CHD), more frequently among those with a syndrome than ones without and also among boys compared to girls. This information can help in designing treatment plans to individuals at risk for BMP.
- Threshold for treating the CHD children in DGA seems lower compared to general population, when 11% were treated in DGA vs. 0.2% in general population. This emphasises the need for promoting means of CBT/BT for children with CHD.
- No association was found between BMP and the number of re-operations or length of the post-operative recovery. On the other hand, an association between BMP and present and past caries experience existed. Dental care of CHD children should focus at health promotion and preventive care.

## Data Availability

Not applicable.
